# 

**Published:** 1836-07

**Authors:** 


### CONTENTS OF No. III.

OF THE

### BRITISH AND FOREIGN MEDICAL REVIEW.

### PART FIRST.—Analytical and Critical Reviews.

PAGE

ART. I.—A further Inquiry concerning Constitutional Irritation, and the Pathology of the Nervous System. By BENJAMIN TRAVERS, F.R.S., senior Surgeon to St. Thomas's Hospital, &c. . . . . . . 1

ART. II.—Leçons de Clinique Médicale, faites à l'Hôtel-Dieu de Paris par le Professeur A. F. CHOMEL, recueillies et publiées sous ses yeux par J. L. GENEST, D.M.P. (Fièvre Typhoide.) . . . . . 33

Clinical Lectures delivered by Professor CHOMEL at the Hôtel Dieu in Paris, (Typhoid Fever.) Collected and published by J. L. GENEST.

ART. III.—A Practical Treatise on Midwifery; containing the Result of 16,654 Births, occurring in the Dublin Lying-in Hospital, during a Period of Seven Years, commencing November, 1826. By ROBERT COLLINS, M.D., late Master of the Institution . . . . . . . 71

ART. IV.—Mémoire sur une Manière nouvelle de pratiquer l'Opération de la Pierre. Par le Baron DUPUYTREN. Terminé et publié par L. J. SANSON, Chirurgien de l'Hôtel Dieu, &c., et par L. J. BEGIN, Chirurgien en chef à l'Hôpital Militaire de Strasbourg, &c. . . . . 98

Memoir on a new Method of Cutting for the Stone. By BARON DUPUYTREN. Completed and published by L. J. SANSON and J. L. BEGIN.

ART. V.—An Essay on the Laryngismus Stridulus, or Croup-like Inspiration of Infants. To which are appended, Illustrations of the general Principles of the Pathology of Nerves, and of the Functions and Diseases of the Par Vagum and its principal Branches. By HUGH LEY, M.D. &c. . . . 112

ART. VI.—A Treatise on the more obscure Affections of the Brain, on which the Nature and successful Treatment of many Chronic Diseases depend. Being the Gulstonian Lectures, delivered at the College of Physicians, in May, 1835. By A. P. W. PHILIP, M.D., F.R.S.L. & E., &c. . . . . 129

ART. VII.—A Theoretical and Practical Treatise on the Diseases of the Skin. By P. RAYER, M.D., Physician to the Hôpital de la Charité, &c. With an Atlas. The Second Edition, entirely remodelled . . . . 147

ART. VIII.—Medico-Chirurgical Transactions; published by the Royal Medical and Chirurgical Society of London. Volume the Nineteenth . . 158

1. Cases of Fracture of the Neck of the Femur, with the Appearances observed after Death. By Mr. HOWSHIP . . . . 159
2. Cases of Warty Tumours in Cicatrices. By CÆSAR HAWKINS, Esq. . 161
3. A Case of Abdominal Tumour. By Mr. HOWSHIP . . 163
4. A Case of Pulmonary Phlebitis. By Dr. R. LEE . . ib.
5. On Ulceration of the Cartilages of the Joints, &c. By Mr. H. MAYO . ib.
6. Singular Laceration of the Peritoneal Coat of the Uterus. By Mr. PARTRIDGE 164
7. On the Chemical Constitution of Calcareous Tumours of the Uterus, and other Parts. By JOHN BOSTOCK, M.D. F.R.S. &c. . . . ib.
8. On Fibro-calcareous Tumours and Polypi of the Uterus. By Dr. R. LEE 165
9. Further Remarks on the Ulcerative Process. By C. ASTON KEY, Esq. . 166
10. Mr. COCK and Mr. THURNAN on the Ear . . . 167
11. Dr. SEYMOUR on Mental Derangement . . . ib.
12. Cases and Observations illustrative of Diagnosis when Adhesions have taken place in the Peritoneum; with Remarks upon some other Morbid Changes of that Membrane. By RICHARD BRIGHT, M.D. F.R.S. &c. . ib.
13. On the Medicinal Properties of Creosote. By J. ELLIOTSON, M.D. &c. 168

### PART SECOND.—Bibliographical Notices.

PAGE
ART. I.—On Bloodletting. By JAMES WARDROP, M.D. &c. . . 171
ART. II.—Klinische Mittheilungen, von Dr. F. A. G. BERNDT, Königl. Geheimen Medizinal Rathe, u. s. f. . . . . . 176
Clinical Communications, by Dr. F. A. G. BERNDT.
ART. III.—Remarks on the Unity of the Body, as illustrated by some of the more striking Phænomena of Sympathy, both Mental and Corporeal, with a view of enlarging the Grounds and improving the Application of the Constitutional Treatment of Local Diseases. By GEORGE MACILWAIN, Esq., &c. . 180
ART. IV.—Statistical Inquiry into the present State of the Medical Charities of Ireland: with Suggestions for a Medical Poor-Law, by which they may be rendered much more extensively efficient. By DENNIS PHELAN, Esq., &c. . 183
ART. V.—Elémens de Chimie. Par E. MITSCHERLICH, Professeur de Chimie à Berlin, &c. Traduits de l'Allemand sur la dernière Edition, par M. B. VALERIUS, de l'Université de Gand . . . . 188
ART. VI.—Illustrations of the Comparative Anatomy of the Nervous System. By JOSEPH SWAN, Esq. . . . . . . . 192
ART. VII.—The Anatomy of the Regions interested in Surgical Operations performed upon the Human Body; with occasional Views of the Pathological Conditions which render the Interference of the Surgeon necessary. In a Series of Plates the size of life. By J. LEBAUDY, M.D. Parts I. II. III. . 196
ART. VIII.—Guy's Hospital Reports. No. II. April, 1836. Edited by GEORGE H. BARLOW, M.A. and JAMES P. BABINGTON, M.A. . . . 198
St. Thomas's Hospital Reports. By JOHN F. SOUTH, Assistant Surgeon. No. II. and III. January, 1836 . . . . ib.
ART. IX.—System der Chirurgie. By P. F. VON WALTHER, Doctor der Philosophie, Medicin und Chirurgie, &c. . . . . 202
ART. X.—A brief Memoir of Sir WM. BLIZARD, Knt., F.R.S. L. and E., Surgeon and Vice-President of the London Hospital. Read before the Hunterian Society, 7th October, 1835; with additional Particulars of his Life and Writings. By WM. COOKE, Member of the Royal College of Surgeons, &c. . . 205
ART. XI.—Formulaire pour la Préparation et l'Emploi de plusieurs nouveaux Médicaments. Par F. MAGENDIE. Huitième Edition, revue et augmentée . 207
A Formulary for the Preparation and Medical Administration of certain new Remedies. Translated from the French of M. MAGENDIE, with Annotations and additional Articles, by JAMES MANBY GULLY, M.D. . . ib.
ART. XII.—A new Practical Formulary of Hospitals of England, Scotland, Ireland, France, Germany, &c. Translated from the new French Edition of MM. MILNE EDWARDS and P. VAVASSEUR, and considerably augmented, by MICHAEL RYAN, M.D. . . . . . 210
ART. XIII.—Philosophical Transactions of the Royal Society of London, for the year 1835. Parts I. and II. . . . . . 212
Proceedings of the Royal Society. Nos. 18—23. From November 20, 1834, to February, 1836 . . . . . . . ib.
ART. XIV.—Das Streckfieber und dessen Behandlung, im Umriss dargestellt. Von Dr. G. C. REICH, Professor der Medicin, &c. . . . 219
An Outline of the History and Treatment of the Fever of Growth. By Dr. G. C. REICH, Professor of Medicine, &c.
ART. XV.—Eupædia; or, Letters to a Mother on the watchful Care of her Infant, in reference to Diet, Clothing, Air, Exercise, Medicine, &c. By A PHYSICIAN 221
ART. XVI.—On Dropsies connected with suppressed Perspiration and coagulable Urine. By JONATHAN OSBORNE, M.D. . . . . 222
ART. XVII.—On Diseases of the Hip-Joint; with some Observations on Deformities of the Chest. By WM. COULSON, M.R.C.S. . . . 231

PAGE

Art. XVIII.— THE FOREIGN JOURNALS. No. III.

Danish Journals:

4. Samlinger til den Danske Medicinal Historie, udgivne af Dr. J. D. Herhodlt og Dr. F. V. Mansa . . . . 231
Collections in the Medical History of Denmark, &c.

The German Journals:

9. Medicinische Zeitung. Herausgegeben von dem Verein für heilkunde in Preussen. (Medical Gazette, &c.) . . . 232
10. Zeitschrift für die Ophthalmologie. Herausgegeben von Dr. F. A. von Ammon, Professor an der Chirurgisch-medicinischen Akademie zu Dresden . . . . . . ib.
Journal of Ophthalmology. Edited by Dr. F. A. von Ammon.

American Journals:

4. The American Journal of Pharmacy, published by authority of the Philadelphia College of Pharmacy. Edited by R. E. Griffith, m.d. 233

Colonial Journals:

1. Revista Medica Fluminense, publicado pela Sociedade de Medicina do Rio de Janeiro . . . . . . 234
The Rio Medical Review, published by the Medical Society of Rio Janeiro ib.

### Part Third.—Selections from Foreign Journals.

### ANATOMY.

On the Arteries of the Corneal Conjunctiva. By Dr. Römer, of Vienna . 255

### PHYSIOLOGY.

On the Power by which the Head of the Thigh-bone is retained in its Socket. By Dr. Eduard Weber . . . . . . 236

### MEDICINE,

#### PATHOLOGICAL, PRACTICAL, AND THERAPEUTICAL.

On Thymic Asthma. By Dr. Hirsch, of Kœnigsberg . . . 237
Illustrations of the Diseases produced in Man by the Affection termed Glanders in Horses. By Dr. Alexander, of Utrecht; Dr. Berra, of Mantua; and Dr. Fenelli, of Ostiani . . . . . . . 241
New Mode of administering Balsam of Copaiba. By M. Ratier . . 243
On the Effects of Indigo in Epilepsy and other Spasmodic Affections. By Dr. Roth, of Mayence . . . . . . . 244
Case of unusually prolonged Sneezing, cured by Snuff. By Dr. Bauwens . 245
The Magnet, a Remedy for Gout . . . . . . 246
On the Relations between the State of the Pulse, Respiration, and Temperature of the Body, in Diseases. By Dr. Al. Donné . . . ib.
On the Presence of Worms in the Air-Passages. By L. Aboussohn . . 249
Salutary Effects of Smallpox. By L. Polletti, m.d. . . . 250
Report on Vaccination in France during 1833. By M. Gerardin . . 251
On Carburet of Sulphur in Rheumatism. By Professor Otto, of Copenhagen . 252

### SURGERY.

On the Treatment of Inflammation of the Testicle by means of Compression. By J. C. F. Fricke, Surgeon to the General Hospital in Hamburg . . 253
On Fractures of the Inferior Extremity of the Radius. By G. Goyraud, m.d. of Aix. (*With Woodcuts.*) . . . . . . 256
Surgical Observations. By Professor Chelius, of Heidelberg . . 259
On the Use of Belladonna as a Topical Application in Retention of Urine, Spasmodic Contractions of the Uterus, and in Strangulated Hernia. By M. Gerard, M. Carré, and Pietro Porta, m.d. . . . . 261

PAGE

On the Influence of Atmospheric Heat in the Cure of Wounds and Ulcers. By M. JULES GUYOT, D.M.P. . . . . . 263
On the Treatment of White Swellings. By M. LISFRANC . . 266
Chlorine Gas as an Injection for the Cure of Hydrocele. By M. DECONDÉ . 267
On Traumatic Ophthalmia. By Dr. SCHINDLER . . . ib.
On Foreign Bodies adhering to the Cornea, and the Manner of removing them. By Dr. SCHINDLER . . . . . . 269
On the Power of Nature in curing severe Diseases of the Eye. By Dr. LORCH . 270

### MIDWIFERY.

Case of Cæsarean Operation performed three times successfully on the same Individual. By Dr. MICHAELIS, of Kiel . . . . ib.
Report of Cases at the Lying-in Hospital at Dresden, during the years 1833 and 1834 273
On the Acidity of the Catamenial Blood; and on the Theory of its Origin. By M. C. RETZIUS, M.D., of Stockholm . . . . 274
A new Diagnostic Sign of Pregnancy. By Professor KLUGE, of Berlin . 275
Use of Ergot in inducing Abortion. By Dr. WEIHE . . . 276

### MEDICAL STATISTICS.

Statistical Researches on Calculous Diseases. By Dr. CIVIALE . . ib.
On Suicide in the Canton of Geneva. By M. PREVOST . . 277
Statistical Report of the Cholera in Italy, in the year 1835; containing the approximative Results, from its invasion to its cessation, in the Cities where it has chiefly prevailed . . . . . . . 279
Vaccination in Austria . . . . . . 280
Revaccination in the Prussian Army in 1834 . . . . ib.

### CHEMISTRY, PHARMACY, &c.

Detection of Sugar in the Blood in Diabetes, and on the best Mode of separating the same Substance from the Urine. By FELICE AMBROSIONI, of Milan . 281
On the Effects of Boiling Meat and Vegetables. By M. CHEVREUL . . 282
Simplified Method of obtaining Creosote. By ANT. GIORDANO . . 283

### TOXICOLOGY.

Experiments on the Action of Oxalic Acid. By J. W. ARNOLD . . 284

---

### PART FOURTH.—Medical Intelligence.

On the PRESENT STATE of MEDICINE in DENMARK. By C. OTTO, M.D., Professor of Medicine in the University of Copenhagen.—First Report: On the MEDICAL INSTITUTIONS and the MEDICAL PROFESSION . . . 285
The Siamese Twins . . . . . . 299
Provincial Medical and Surgical Association . . . . 300
Eastern Provincial Medical and Surgical Association . . . ib.
A Literary (Medical) Curiosity . . . . . 301
New Magazine of Zoology and Botany . . . . ib.
The Jacksonian Prize . . . . . 302
Fothergillian Prize Essays for 1837 and 1838 . . . . ib.
OBITUARY.—JOHN FLETCHER, M.D. . . . . ib.
NATHAN DRAKE, M.D. . . . . 305
Dr. WALSHMAN . . . . . ib.
DAVID HOSACK, M.D., F.R.S. L. & E. . . 306

---

LIST of BOOKS received for Review . . . . . ib.

